# Where do UK clinicians find information at the point of care? A pragmatic, exploratory study

**DOI:** 10.1186/s12875-024-02627-7

**Published:** 2024-10-23

**Authors:** Margaret McCartney, Kate Connolly, Frank Sullivan, Carl Heneghan, Elijah Yu Heng Ho, Brid Hendry, Charlotte Salisbury, Sam Offer, David Nunan

**Affiliations:** 1 School of Medicine, University of St Andrews, St Andrews, KY16 9TF UK; 2 Nuffield Department of Primary Care Health Sciences, University of Oxford, Radcliffe Primary Care Building, Oxford, Observatory Quarter, Woodstock Rd OX2 6GG UK; 3 University of Glasgow Medical School, Wolfson Medical School Building,University Ave, Glasgow, G12 8QQ UK

**Keywords:** General practice, IT, Education

## Abstract

**Aim:**

To describe where clinical information is contemporarily and commonly found in UK primary care, what is favoured by clinicians, and whether this is (1) publicly funded (2) has commercial potential conflicts of interest.

**Design and setting:**

A mixed methods study, consisting of (1) site visits to general practices in Scotland, (2) online questionnaire, focused on UK general practice (3) analysis of materials cited by professionals.

**Methods:**

Data about sources of clinical information used was obtained verbally, visually and via search histories on computers from visits. This was used to inform a questionnaire in which primary care clinicians in the four nations of the UK were invited to participate. This obtained data about the information sources used and preferred by clinicians. This information was searched for data about funding and conflicts of interest.

**Results:**

Over 2022, four practices were visited. 337 clinicians, 280 of whom were general practitioners completed an online questionnaire. 136 different resources were identified. These were mainly websites but sources of information included colleagues, either in practice or through online networks, apps, local guidelines, health charities, and learning resources aimed at GPs. Of these, 70 were not publicly funded, and were a mixture of membership organisations, charities, or sponsored venues.

**Conclusions:**

Primary care clinicians obtain information for themselves and patients from a wide variety of sources. Funding is from a variety of sources and some contain advertising and/or sponsorship, risking commercial bias.

**Protocol:**

Pre-published at https://osf.io/wrzqk.

**Supplementary Information:**

The online version contains supplementary material available at 10.1186/s12875-024-02627-7.

## Background

General practitioners use a large volume of disparate information in clinical practice. The public sector provides some of this information. Information sources currently preferred and commonly used by primary care clinicians has not been recently described, and nor has their potential conflicts of interest and bias.

### How this fits in

Much of GP knowledge is recognised as ‘tacit’ and is often unwritten or used via ‘mindlines’. The growth of the internet and rise of different working patterns, including remote and asynchronous work changed the opportunities GPs have in finding information. GPs have a rich knowledge of online resources to assist them. Publicly funded provision does not appear to satisfy all needs, and commercially funded information sources are also used. This may attract some risk of biased information.

## Introduction

General practitioners routinely generate multiple questions while consulting with patients [1]. These relate to diagnosis, treatment, ongoing management, and to locate or refer patients to sources of information. However, international evidence suggests that many of these questions are never answered. Only a minority are fulfilled ‘just in time’ within the consultation. Although information -seeking does occur outside the consultation, this occurs in only a small amount of cases, and takes large amounts of time to complete [2]. There is wide variation in the amount and type of clinical information sought by GPs [3, 4]. Multiple resources, many with public funding through the NHS, and others, through professional societies, have been created to try and fill these gaps. However it is uncertain whether these achieve this aim. While ‘package’ electronic resources and synthesised summaries of evidence have been previously shown to answer many questions, these are of variable quality [5, 6]. Few studies address issues related to the use of clinical information resources in multidisciplinary team working, or investigate clinical resources used by members of the primary care team other than by doctors [7]. The recognition of the need to share information with patients in order to make value-based, shared decisions has been exemplified in Scotland within the ‘Realistic Medicine’ movement. Shared decision aids have been suggested as a method to achieve this. However these do not appear to make large changes to outcomes, are not frequently used in practice and improve process rather than patient outcomes [8]. Other interventions which have been developed to help primary care staff make ‘just in time’ rapid decisions include decision support tools to assess the risk of undiagnosed cancer. However these are also uncommonly used [9]. NHS Scotland has access for primary care staff to access online depository of online journals, however only 2.5% of GPs are registered with OpenAthens usernames (personal communication.) A large amount of effort and resources is used to create information for primary care staff. Given the volume of unanswered questions, the effectiveness of these resources in meeting the needs of primary care staff is unknown.

The concept of ‘mindlines’ was generated in 2004 by Gabbay and le May as an explanation of how information is sought and used by general practitioners [10]. They found that questions asked by GPs were not typically answered by guidelines or traditional book resources. Instead they described ‘collectively constructed’, practice based, accumulated knowledge, often informally shared between staff. Gabbay and le May, researching in the infancy of widespread internet use found that it was uncommonly used in practice. While continuing professional education still draws on a wide variety of resources, the wide availability of the internet has made numerous and different resources available in real time to answer clinical questions at the point of care [11]. GPs do not follow aspects of clinical guidance, with general practitioners citing clinical judgement, a mismatch between evidence available to answer questions, the incorporation of patient values, and workload as reasons why [12–15]. Multiple types of information are incorporated when making clinical decisions, and strategies to update ‘mindlines’ with current knowledge have been posited to improve clinical care, although these have not been tested [16]. Guideline resources aimed at general practitioners do not often answer the questions GPs ask in clinical practice [17]. Currently, we do not know where primary care staff look for information in real time.

Additionally, concerns have been raised about the funding arrangements for educational resources aimed at doctors. Associations have been noted between exposure to pharmaceutical company information and lower quality prescribing [18]. Doctors have been found not to detect bias in industry funded educational materials, “*unsurprising given the depth to which industry messaging has become ingrained*” [19]. Case studies demonstrate that medical education has been used to drive off label prescribing of potentially addictive drugs [20]. Use of internet based resources has the potential to widen the ability of sponsored education to reach doctors, including doctors who would normally avoid traditional educational events or pharmaceutical representatives. The current potential for educational resources to be affected by funding bias is unknown.

## Methods

All general practice sites in NHS Fife and Greater Glasgow and Clyde Health Board area were invited to participate. Further information and a consent form was sent to the practices indicating interest in participation.

Qualitative data: Sites who agreed were visited by a member of the research team and information about resources used by primary care clinicians was gathered. This consisted of: verbal, scribed discussion between the visiting researcher and staff members, (narrating resources used), photography (posters, books, or desk aide memoirs), online resources (including e.g. apps on mobile devices) and printouts (from computers of clinical search histories used). Irrelevant information, non clinical information, and information about access to local services (e.g. sports facilities or support for domestic violence) was excluded. Photographs were deleted once relevant data had been obtained.

Information about each resource as generated from each practice was entered into an Excel spreadsheet and the following extracted: type of information, date of publication, and funding source.

Integration of data: These data sources were used to develop a questionnaire using Qualtrics. The questionnaire sought to discover what information sources were used and preferred by primary care clinicians. The information sources identified at practice visits were offered as ‘tickbox’ options for participants. Information was also obtained on additional, publicly funded, sources of information in Scotland, Wales and Northern Ireland by searching each nations’ staff platforms and national educational organisations. This was supplemented by discussions with clinicians known to the research team from each of these nations to ensure that no large publicly funded information sources generated by each of the four nations were missed. These were included as ‘tickbox’ options and available dependant on the geographical location of participants.

Quantitative data: The questionnaire (SUPPLEMENT 1) was circulated to primary care clinicians via social media using snowballing techniques, but also via the Royal College of General Practitioners (UK) newsletter. Clinicians were asked to describe which of the popular resources and publicly funded resources we had located they used. Clinicians were asked to rate ‘*This is a favourite’* as an indication of their most favoured resources, with no limit on the number. At the end of the questionnaire clinicians were invited to use free text to “*Please tell us your most useful/favourite/helpful clinical resources (as many as you like)*”. Clinicians were asked to name as many ‘favourite’ resources as they wished in a free text box. These were tabulated by hand into an Excel spreadsheet and frequency analysis performed. No other demographic information was requested, as the aim was to obtain broad information in as wide a participatory group as possible, aiming to allow efficient completion, given the workforce crisis in general practice and the demands on staff time.

Websites of resources named by participants were then reviewed for information about whether they received public funding via the NHS or Government, or had private funding or non-publicly funded arrangements. This was usually via ‘About Us’ links. The website was searched for clear statement of public funding and recorded if present. Information about any other type of funding was reviewed by a second researcher, and categories were iterated with the results. If a funding statement inferred non-public funding, a specific search was then undertaken. This gathered information relating to the organisations’ position on advertising and sponsorship, or statements welcoming these, focussed on companies manufacturing items that could be prescribed, e.g. pharmaceutical or medical device manufacturers. Data relating to whether the websites required a subscription, or academic access, was extracted. If no information about funding could be located, email communication was sent enquiring and the results recorded.

The top 10 ‘favourited’ information sources which had elements of patient facing information (either aimed at patients, or with patient information contained and authored by the website) were analysed against the DISCERN tool and JAMA Benchmark tool to assess quality (SPREADSHEET 1) [21, 22]. Google images/Google were excluded.

### Protocol deviations

This research was conducted at a time of crisis in supply and demand inequity in UK general practice as it emerged from the covid-19 pandemic. It had been planned to visit more practices and obtain wider information about sources used before including this in the questionnaire. Because the response rate for practice visits was low, personal communication with clinicians and information specialists known to the research team was used to locate publicly funded websites for questionnaire inclusion as a pragmatic response. With appreciation of time pressures on staff, rather than using formal qualitative interviews to obtain data in practices, we scribed verbal information during visits about information sources, used photographs to record information, thus generating maximal information for inclusion in the questionnaire.

It had been intended to analyse the data generated from practice visits from analysis of history on search engines, but these often did not contain historical information beyond 7 days.

It was intended to analyse the top 10 websites containing public-facing information as described on the questionnaire, but 12 were included, as all were mentioned more than once on the survey.

## Results

### Practice results

51 general practice sites in NHS Fife were emailed directly, and an email newsletter including information about this study was sent to 226 practices in Glasgow. Of these, 8 agreed to participate, but four were unable to due to workload and/or time constraints. One of these (practice 5) did supply some information verbally but was not visited. All were in urban areas.

Types of resources used included books, posters, printouts, apps via personal mobile devices and websites. Only resources containing clinical information were included. All practices visited stated that they also asked colleagues for advice, and valued clinical information and clinical discussion from them. The range of resources identified varied from 6 to 35, average 26.2, per practice. Academic reference books (such as Oxford Handbooks of Clinical Medicine) were excluded from totals and further analysis.

An Excel spreadsheet was generated to include the resources identified. New information from each practice was added. No attempt was made to estimate the most frequently visited resources. Resources used were amalgamated, for example, information generated by the different parts of the National Institute for Health and Care Excellence, or the Scottish Intercollegiate Guidelines Network, or local health boards were counted only once under each headline. Two exceptions were (1) the Clinical Knowledge Service, which is commissioned by and funded by NICE but has distinct development processes and (2) Travax, a travel information website funded by Public Health Scotland. These were analysed separately. Websites which acted as conduits to other resources (e.g. online library services) which did provide their own authored clinical material were excluded from this analysis.

### Questionnaire results

The questionnaire was completed by 337 clinicians, 280 of whom were general practitioners. Other clinicians included nurses, pharmacists, physiotherapists and optometrists. The majority worked in England (152), Scotland (112), Wales (18) and Northern Ireland (10).

The majority of clinicians in Scotland stated they never used the publicly funded Right Decision Service, Turas Learn, or the Knowledge Network Scotland. However SIGN (Scottish Intercollegiate Network) guidelines were used by over half of participants a few times per year or more. Similarly, in Northern Ireland, most never used the National Healthcare Library, however GP Northern Ireland was stated to be used by most participants a few times a year or more. In Wales, over half of participants never used Learning@Wales and over 90% never used Wales Mental Health in Primary Care (SUPPLEMENT 2).

Overall, of the list presented to clinicians (SUPPLEMENT 3), clinicians rated the BNF (British National Formulary) and NICE guidelines as favourites, followed by Patient Info (a platform run by a private company which has access links through a GP computer operating system, EMIS) and DermNet NZ (a free to access dermatology image library from New Zealand) and GP Notebook (a privately owned company) as their top five ‘favourites’. The top five resources described as being accessed most frequently were TripDatabase, MDCalc, Patient Info, GPNotebook, and DermNet NZ. The resources described most frequently as being ‘never’ accessed were the Cochrane Library, TripDatabase, MIMs, FourteenFish, and PubMed.

Free text was entered by 221 participants: two made comments which did not include any additional named resources. Excluding these, a total of 639 resources were described, ranging between 1 and 13 with an average of 2.9. Participants listed 136 different resources in this section. (SPREADSHEET 2**)**. Resources were condensed, for example, all versions of the British National Formulary (BNF) were included under ‘BNF’ (i.e. the web app, children’s version, and book). Clinicians used free text to describe other issues with accessing resources, for example, the inaccessibly of previously used and liked websites (for example, due to health board choices of computer systems and subsequent browser incompatibility). Participants occasionally wrote that some resources were ‘best’ or ‘used every day’. Other respondents mentioned speed, illustrations, and patient-centred information as valuable, for example “can print specific physio information etc” and “often direct patients here”.

Some guidelines, specific to a locality, appeared popular with individuals from a mixture of geographies. Because this may have indicated particularly useful resources, those named were retained separately. However, if participants mentioned ‘local resources’ without further clarification (e.g. ‘local formularies’), these were combined.

The most frequently named resources were the CKS (Clinical Knowledge Service, named by 100 respondents), the British National Formulary (BNF), named by 49 respondents) GP Notebook (34 respondents), DermNet (22 respondents), the search engine Google (including Google Images and Scholar, 21 respondents). The top 10 most popular cited sources are listed in Table 1.

**Table 1:** Types of sources identified

Resource	Number of citations	Organisation Type	Funding Source
Clinical Knowledge Service (NICE)	100	Public	UK Government
BNF	49	Private company	Sales
Local guidelines/formularly/intranet	40	Public	Local health boards/central NHS
GP Notebook	34	Private company	Advertising
Primary Care Dermatology Society	27	Membership, Charity, Company	Pharmaceutical industry sponsorship
Red Whale	26	Subscription	Independent
Dermnet NZ	22	non-public, includes volunteering	Sponsorship/advertising/donations
Patient UK	18	Private company	Sponsorship, advertising
Google (images, scholar)	21	Private company	Advertising
NICE Guidelines	18	Public	Government
NB Medical	16	Private	Pay-for

Funding arrangements were noted as not always immediately apparent, requiring searching on charity accounts, Disclosure UK, or in the ‘small print’ of websites. Of the 136 listed resources, 66 were publicly funded.

The 70 non publicly funded organisations were further analysed (SPREADSHEET 3).

These were found to be a mixture of charities (6), charities who were also membership associations (11), charities who were membership associations (1), charities who were membership associations and also operated as companies (1), membership associations (4), membership associations also functioning as trade unions (1); companies also operating as trade unions (1), companies also operating as private organisations (4) and privately owned companies (41). One private company was supported by the NHS (CPD Connect, a paid-for continuing professional development organisation facilitated by NHS Education for Scotland). Privately owned organisations included those with a non-profit, open access stance (e.g. Buku). Charities in the UK must have charitable aims (defined in law), be exclusively established for the public benefit, cannot make profit, and can include professional and patient organisations.

12 companies were assessed as having no visible advertising or sponsorship. These included publishing and medical educational companies (two of whom specifically disavowed commercial sponsorship). These also included the Merck Manual, published by Merck, a pharmaceutical company, and the British National Formulary, which contains no adverts, but is funded by sales of the BMJ (which contains pharmaceutical advertising) and the Pharmaceutical Press. 13 contained evidence of adverts, 10 were sponsored sites with advertising, 1 was FOAM (free online medical education) with sponsorship, and 1 was FOAM with adverts. 4 operated as subscription platforms, some with free content. 2 websites belonged to privately operating (i.e. non National Health Service) clinics. 2 were free with Athens (academic library) subscriptions. 1 was of an uncertain funding model (theNNT), and contact with the owner via email for clarification received no reply.

DISCERN and JAMA benchmark tools were used on 12 rather than the pre specified 10 sites, thus including all resources mentioned more than once. These generally scored highly, with all sites considered to supply clear, relevant information which achieved their aims. However, for 5/12 sites, it was considered ‘unknown’ whether the information could be biased, mainly on the basis of sponsorship, advertising, or some alternatives to intervention recommended being absent.

## Discussion

The wide range of resources cited indicates how primary care clinicians draw extensively on both publicly and privately funded resources in order to meet their, and their patients’ needs. This included local resources, such as guidelines and networks, and national resources, such as the Clinical Knowledge Network. Clinicians also used internationally based resources, such as Dermnet NZ (originating in New Zealand) and MD Calc. Gabbay and le May observed how general practitioners utilised each other as a resource in 2004, noting that relevant knowledge was not always held in written form [10]. Studies on opinion leaders in primary care show that this is often within their own practice [23]. The need for information sharing between peers remains evident, with GPs stating that ‘colleagues’ remain an important source of information. In the survey, clinicians also noted that Facebook and Whatsapp private groups served as a way to communicate timeously with their peers. This may be of particular note given the changes in working patterns over the Covid-19 pandemic, with GPs now able to work from home or online. In free text comments, some GPs noted that they had been forced to change their uses of online information for patients because of health board decisions about software use, making some previously valued websites (for example, Uptodate, a clinical education website) unobtainable. Pragmatism and adaptability was evident. Several GPs commented that they used Google searches frequently and assessed information presented for suitability for themselves or their patients for needs at that time, and described how they would search for a term, adding ‘NHS’ into Google to make better resources appear first, e.g “*fastest with patient in the room*”. Several clinicians described how they would use Google images searches to discuss and explain appearances with patients. The Clinical Knowledge Network, which is publicly funded and based on the National Institute for Health and Care Excellence (NICE) website, was particularly lauded, with free text responses such as ‘the dream resource”, and “I do love” it. Distributions of some resources may have reflected subscription models, for example, FourteenFish appearing as a highly ranked favourite but was also highly ranked as never used.

Strengths of the study include the number and the detail of responses to the survey, the detailed examination of funding of websites, and the wide range of resources examined. This enabled an overview of contemporary use of resources in primary care in the UK. There are several limitations. The participants were already online, meaning that clinicians with who used the internet in limited ways would be less likely to participate. A small number of practices were visited from a limited geographical area, and it is possible that other practices or areas use very different resources. No claims can be made about the relative use of these resources, as this information was not obtained. Regardless of whether a website was sponsored or not, this does not necessarily infer that clinicians use biased information, as the clinician has discretion over how to interpret and use it. Further, the use of the website rating tool DISCERN and JAMA benchmark do not examine how well evidenced advice is. For example, we noted that one website examined had incorrectly interpreted information from a cited research paper. There are therefore concerns that these tools may miss key assessment of reliability regardless of how well resources are constructed. Nor does a statement of disclosure negate possible bias, meaning that a resource could score highly while having misleading content. Additionally, recall bias may have affected results of the questionnaire.

It is noted that almost half the resources cited by GPs as favourites were not publicly funded. Some publishing organisations were companies, selling subscriptions or educational products. Two such websites, each educational and paid-for by subscription, made explicit statements that they did not have pharmaceutical funding. However, one was noted to carry an educational video authored by a patient organisation which was known to be part funded by industry. More broadly, adverts on websites were generally for health-related products including over the counter and prescription drugs and/or devices. Sponsorship for websites was wide ranging. It included that from pharmaceutical companies, often multiple. For example, the British Association of Dermatologists stated online that “*Sponsorship and exhibition opportunities are available for many of our in-person and virtual events*,* enabling corporate sponsors to target specific audiences…We are also willing to discuss bespoke corporate partnerships relating to communications*,* patient engagement*,* research and more*”. Pumping Marvellous, a patient led charity, was found on Disclosure UK to have been in receipt of over 200,000 UK pounds from AstraZeneca during 2022, including for the production of patient facing materials, awareness campaigns, and validation of a patient questionnaire. The search for locating declarations of interest was noted to be often difficult, with statements in small print, and hard to locate on websites. This time consuming activity may not be possible for busy practitioners.

While the range and depth or resources which clinicians are able to call upon could be regarded as a strength, consideration should be given to commercially funded resources. Advertising prescription only drugs direct to consumers is illegal in the UK but legal to healthcare professionals [24]. A Code of Practice is administered by the Association of the British Pharmaceutical Industry, which contains guidance on what information should be contained in advertisements, for example, indication, classification, warnings, and non proprietary name or active ingredient [25].

Systematic reviews have found associations with advertising and increased prescribing in doctors without net health benefits [26]. The World Health Organisation have said that “*While the evidence is not conclusive*,* what there is all points in the direction of a strong association between reliance on promotion and less appropriate overall use of prescription drugs”* [27]. There is therefore reason to be cautious over the use of commercially sponsored information. However this work was not able to elucidate why clinicians favoured particular sources of information. This might include format, content, accessibility, relevance, and trust. Further work should generate information about what makes certain resources favoured, and should question whether public funding should be used to cover any real or perceived gaps in information without commercial sponsorship. This could include qualitative interviews, which we were not able to perform in this study.

Notably, a recent systematic review found no standard, validated tool to assess the trustworthiness of point-of-care information for health care professionals [28]. It is also noted that the tools used to assess online resources aimed at patients do not directly ask for validation or the reliability of the information it contains. This is also noted in other tools promoted for assessing reliability of information, such as the Health on the Net Foundation [29]. In practice such accuracy may be difficult to judge, as it may require area-specific expertise which is often generated during development processes such as peer review. While some organisations (such as the Clinical Knowledge Service) described a clear pathway to publishing, with internal and external peer review, others did not. Considering the risk of inaccurate information along with the popularity of resources, further work should consider other means of validating information.

Primary care clinicians use a wide variety of information resources in order to fulfil their own and patients needs. The reasons for clinicians using non-publicly funded resources are unknown but may include a mismatch between their needs and publicly funded supply. Validated tools to assess information quality are lacking and should be investigated, given the potential for poor quality information to be in widespread use.

## Electronic supplementary material

Below is the link to the electronic supplementary material.

Supplementary Material 1

Supplementary Material 2
