# Supplementary material for: Where do UK clinicians find information at the point of care? A pragmatic, exploratory study
Source: BMC Prim Care. 2024 Oct 23;25:376. doi: 10.1186/s12875-024-02627-7 (PMC11515728; doi:10.1186/s12875-024-02627-7)
Supplement: Supplementary file 2 — Supplementary Material 2 [file 12875_2024_2627_MOESM2_ESM.docx]

**SUPPLEMENT 1 - QUESTIONNAIRE**

1. What is your job role in primary care?

2. Which country/nation are you working in?

3. Do you use

The Cochrane Library?

The BMJ/BMJ learning?

GP Notebook?

Fourteen Fish?

Patient Info?

the BNF?

NICE guidelines?

Pubmed/Medline?

Trip Database?

Menopause Matters ?

MIMS?

MD Calc?

Dermnet NZ?

For clinicians in Scotland:

the Right Decision Service?

TURAS learn?

the Knowledge Network Scotland

SIGN guidelines?

For clinicians in Northern Ireland:

GP Northern Ireland?

the Northern Ireland Medical and Dental Training Agency?

The National Healthcare Library for Northern Ireland?

For clinicians in Wales:

Wales Mental Health in Primary Care (WMH in PC)?

Learning@Wales?

**SUPPLEMENT 2**

**a)** SCOTLAND


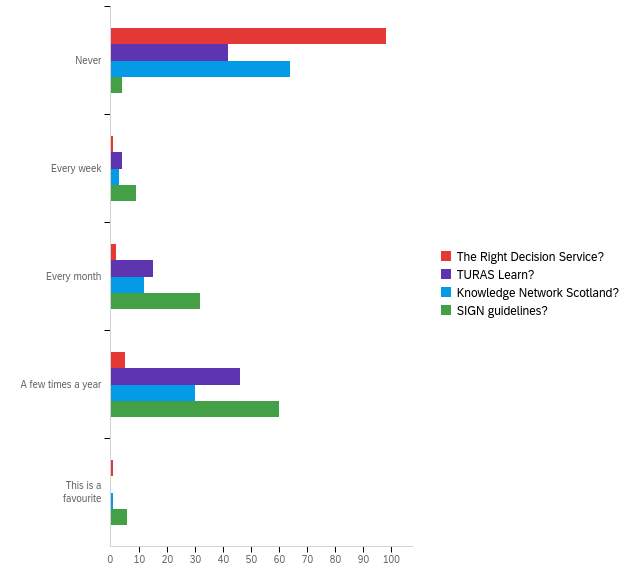


**b) NORTHERN IRELAND**


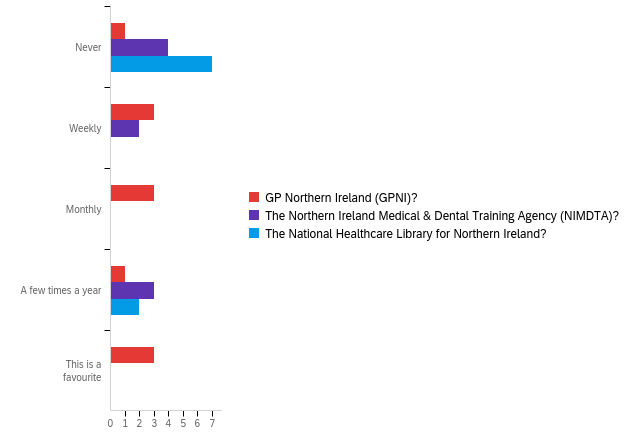


**c) WALES**


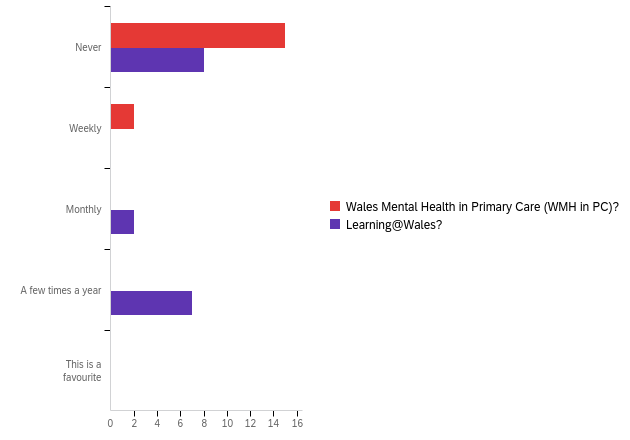


**SUPPLEMENT 3 - RATINGS**


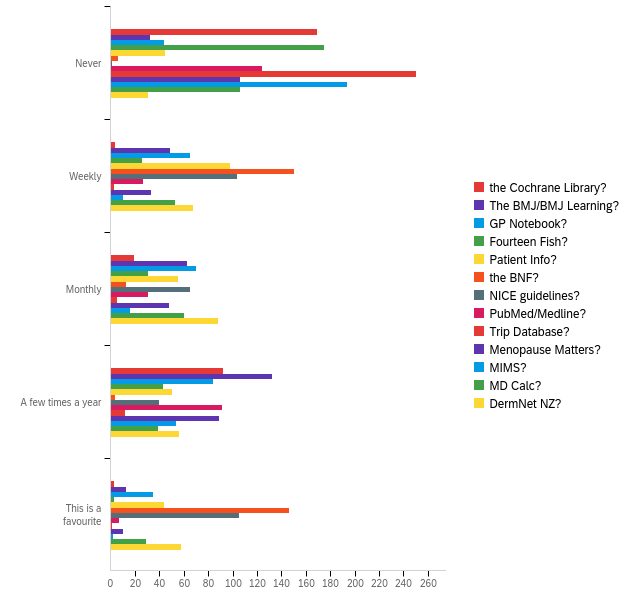


SUPPLEMENT 4

NON PUBLICLY FUNDED RESOURCES


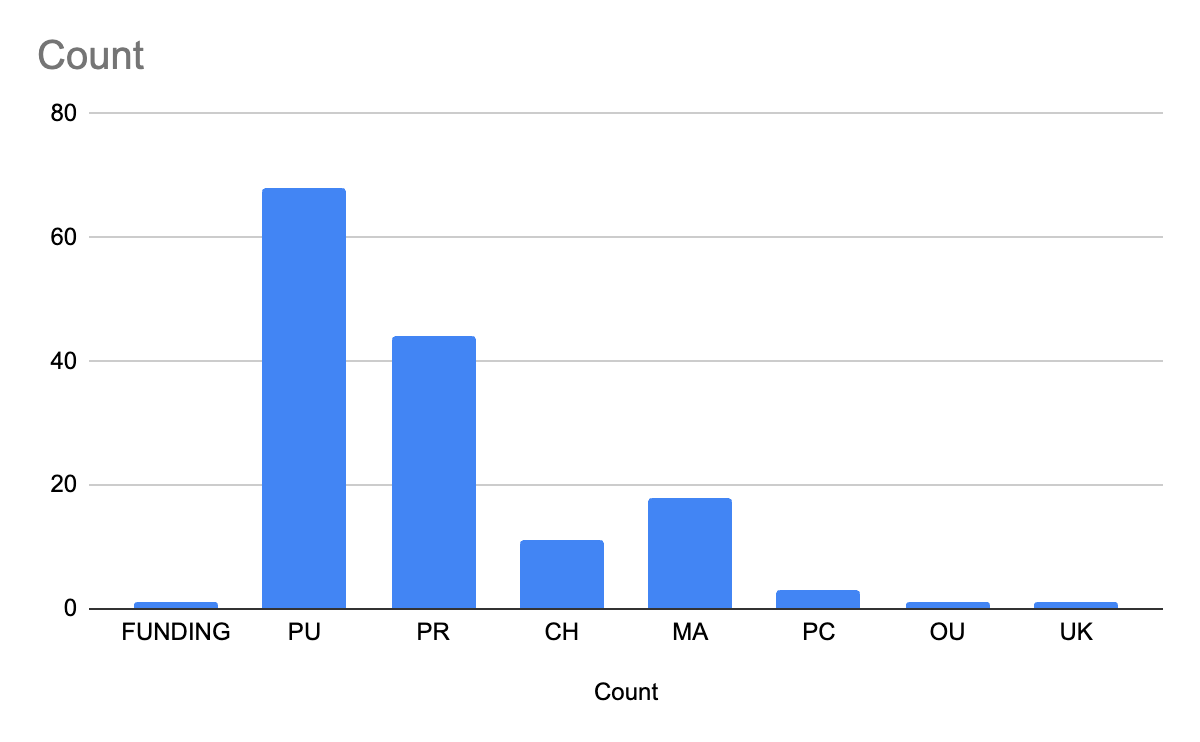


PU- Public

PR - Private

CH- Charity

MA- Membership association

PC- Private clinic

OU-
